# 1,3-Benzothia­zolium tetra­chlorido­aurate(III) tetra­hydro­furan solvate

**DOI:** 10.1107/S1600536809003572

**Published:** 2009-02-06

**Authors:** Tesfamariam K. Hagos, Stefan D. Nogai, Liliana Dobrzańska, Stephanie Cronje, Helgard G. Raubenheimer

**Affiliations:** a Department of Chemistry, University of Stellenbosch, Private Bag X1, Matieland, South Africa

## Abstract

In the crystal structure of the title ionic compound (C_7_H_6_NS)[AuCl_4_]·C_4_H_8_O, the [AuCl_4_]^−^ anion shows a typical square-planar geometry. Numerous weak C—H⋯Cl hydrogen bonds between [AuCl_4_]^−^ and the 1,3-benzothia­zolium units form layers comprised of 24-membered rings in which hydrogen-bonded tetra­hydro­furan (THF) solvent mol­ecules are accommodated. C—H⋯Cl inter­actions between THF and [AuCl_4_]^−^ from adjacent layers result in bilayers. These are further stabilized by π–π inter­actions between the thia­zole and benzene rings [centroid–centroid distance = 3.971 (3) Å], resulting in the formation of a three-dimensional supra­molecular assembly.

## Related literature

For background, see: Hagos *et al.* (2008 ▶). For related compounds, see: Huynh *et al.* (2006 ▶); Yen *et al.* (2006 ▶, 2008 ▶). For bond-length data, see Adé *et al.* (2004 ▶); Asaji *et al.* (2004 ▶); Makotchenko *et al.* (2006 ▶). For related literature, see: Brammer *et al.* (2001 ▶).
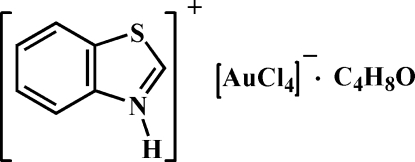


## Experimental

### 

#### Crystal data

(C_7_H_6_NS)[AuCl_4_]·C_4_H_8_O
*M* *_r_* = 547.06
Triclinic, 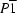

*a* = 7.3213 (7) Å
*b* = 10.3498 (10) Å
*c* = 11.8783 (12) Å
α = 99.331 (1)°
β = 107.579 (1)°
γ = 104.483 (2)°
*V* = 802.75 (14) Å^3^
*Z* = 2
Mo *K*α radiation
μ = 9.95 mm^−1^
*T* = 100 (2) K
0.30 × 0.20 × 0.10 mm

#### Data collection

Bruker APEX CCD area-detector diffractometer
Absorption correction: multi-scan (*SADABS*; Sheldrick, 1997 ▶) *T*_min_ = 0.101, *T*_max_ = 0.371
4957 measured reflections
3504 independent reflections
3325 reflections with *I* > 2σ(*I*)
*R*_int_ = 0.013

#### Refinement

*R*[*F* ^2^ > 2σ(*F* ^2^)] = 0.027
*wR*(*F* ^2^) = 0.064
*S* = 1.05
3504 reflections
175 parameters
1 restraint
H atoms treated by a mixture of independent and constrained refinement
Δρ_max_ = 2.27 e Å^−3^
Δρ_min_ = −1.00 e Å^−3^

### 

Data collection: *SMART* (Bruker, 2001 ▶); cell refinement: *SAINT* (Bruker, 2002 ▶); data reduction: *SAINT*; program(s) used to solve structure: *SHELXS97* (Sheldrick, 2008 ▶); program(s) used to refine structure: *SHELXL97* (Sheldrick, 2008 ▶); molecular graphics: *X-SEED* (Barbour 2001 ▶); software used to prepare material for publication: *SHELXL97*.

## Supplementary Material

Crystal structure: contains datablocks I, global. DOI: 10.1107/S1600536809003572/ng2541sup1.cif

Structure factors: contains datablocks I. DOI: 10.1107/S1600536809003572/ng2541Isup2.hkl

Additional supplementary materials:  crystallographic information; 3D view; checkCIF report

## Figures and Tables

**Table 1:** Hydrogen-bond geometry (Å, °)

*D*—H⋯*A*	*D*—H	H⋯*A*	*D*⋯*A*	*D*—H⋯*A*
N6—H6⋯O14	0.86 (5)	1.87 (5)	2.728 (5)	177 (6)
C5—H5⋯Cl2^i^	0.95	2.65	3.588 (5)	170
C8—H8⋯Cl4	0.95	2.93	3.447 (5)	116
C9—H9⋯Cl4	0.95	3.00	3.498 (5)	114
C10—H10⋯Cl2^ii^	0.95	2.96	3.541 (6)	121
C11—H11⋯Cl2^ii^	0.95	2.90	3.498 (5)	122
C11—H11⋯Cl3^ii^	0.95	2.77	3.639 (5)	154
C15—H15*B*⋯Cl1^iii^	0.99	3.02	3.922 (6)	153
C18—H18*A*⋯Cl4^iii^	0.99	2.91	3.547 (6)	123

Symmetry codes: (i) 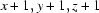; (ii) 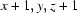; (iii) 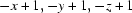.

**Fig. 1.**
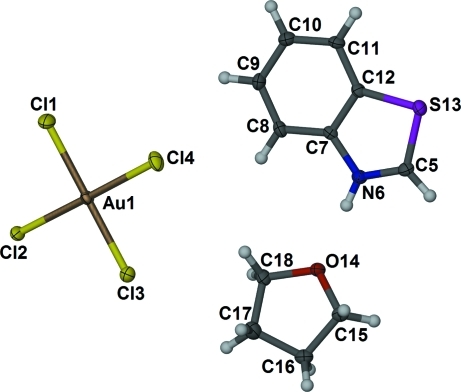
The molecular structure of the title molecule, with the atom-numbering scheme. Displacement ellipsoids are drawn at the 50% probability level.

**Fig. 2.**
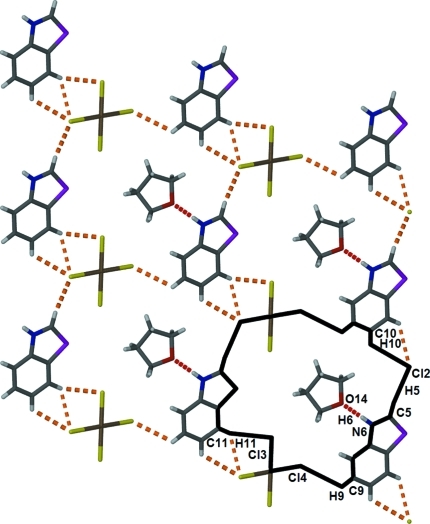
Capped-stick representation showing the formation of layers consisting of R56(24) rings (shown in black). Dashed orange lines represent C—H···Cl hydrogen bonds.

**Fig. 3.**
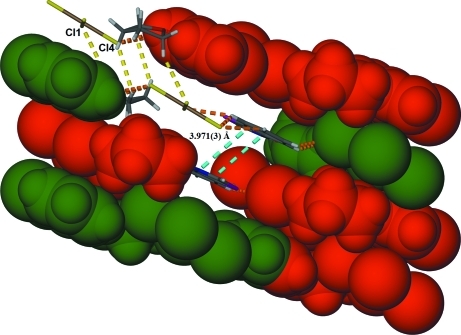
Representation of the bilayers (red-green) with pillar-like connections (yellow dashed lines) extended in the third dimension by π-π interactions (blue dashed lines) viewed down [010].

## supplementary crystallographic information

### Comment

During the course of ongoing studies on the reactions of gold(III) compounds with heterocycles, we have isolated the title ionic compound (I) with a structure resembling that of a 1,3-dimesitylimidazolinium tetrachloro-gold(III) dichloromethane solvate reported earlier (Hagos *et al.* 2008). The asymmetric unit (Fig. 1) consists of a 1,3-benzothiazolium cation, a tetrachloro-gold(III) anion and a tetrahydrofuran molecule. The structural parameters associated with the 1,3-benzothiazolium moiety agree well with reported values, see for example 3-(2-propenyl)-1,3-benzothiazolium bromide (Huynh *et al.* 2006), *N*-benzyl-1,3-benzothiazolium bromide (Yen *et al.* 2006) and 3-n-propyl-1,3-benzothiazolium bromide monohydrate (Yen *et al.* 2008). The anionic part displays a typical square-planar geometry around Au and the Au—Cl distances compare well with previously reported values (Adé *et al.*, 2004; Asaji *et al.*, 2004; Makotchenko *et al.*, 2006). All Cl atoms of [AuCl_4_]^-^ complex participate in the formation of weak C—H···Cl hydrogen bonds (Table 1). Atoms Cl2, Cl3 and Cl4 interact with the 1,3-benzothiazolium cation forming layers consisting of *R*^5^_6_(24) rings in which tetrahydrofuran molecules are incorporated by forming hydrogen bonds O6—H6···N14 with a distance of 2.728 (5) Å (Fig. 2). Further C—H···Cl interactions between THF and [AuCl_4_]^-^ from neighbouring layers (C15—H15B···Cl1 and C18—H18A···Cl4) form pillar-like connections between them, leading to the formation of bilayers. The latter are propagated along [100] by π-π interactions between thiazole and benzene rings [symmetry operation: 1 - *x*, 1 - *y*, 2 - *z*, centroid-centroid distance = 3.971 (3) Å], resulting in a three-dimensional assembly (Fig. 3).

### Experimental

1,3-Benzothiazole (0.10 g, 0.76 mmol) in acetonitrile (5 ml) was treated with HAuCl_4_^.^4H_2_O (0.31 g, 0.76 mmol) in water (5 ml) at room temperature (2.5 h). The reaction mixture was stripped of solvent and extracted with a mixture of dichloromethane and THF (1:1, 150 ml). Then the solvent was removed under reduced pressure to yield a yellow residue. Orange crystals suitable for single-crystal X-ray analysis were obtained from a THF solution layered with n-pentane at 253 K.

### Refinement

H6 atom (for NH) was located in a difference map and refined with a restrained N—H distance of 0.86 (5) Å, and with *U*_iso_(H) = 1.2Ueq(N). The remaining H atoms were positioned geometrically, with C—H = 0.95 and 0.99 Å for aromatic and methylene H, respectively, and constrained to ride on their parent atoms with *U*_iso_(H) = 1.2*U*_eq_(C). The highest peak and deepest hole in the final difference Fourier map are located at 0.88 Å and 0.95 Å from atom Au1, respectively.

### Crystal data

**Table:**

(C_7_H_6_NS)[AuCl_4_]·C_4_H_8_O	*Z* = 2
*M**_r_* = 547.06	*F*(000) = 516
Triclinic, *P*1	*D*_x_ = 2.263 Mg m^−^^3^
Hall symbol: -P 1	Mo *K*α radiation, λ = 0.71073 Å
*a* = 7.3213 (7) Å	Cell parameters from 3347 reflections
*b* = 10.3498 (10) Å	θ = 2.4–28.1°
*c* = 11.8783 (12) Å	µ = 9.95 mm^−^^1^
α = 99.331 (1)°	*T* = 100 K
β = 107.579 (1)°	Block, orange
γ = 104.483 (2)°	0.30 × 0.20 × 0.10 mm
*V* = 802.75 (14) Å^3^	

### Data collection

**Table:**

Bruker APEX CCD area-detector diffractometer	3504 independent reflections
Radiation source: fine-focus sealed tube	3325 reflections with *I* > 2σ(*I*)
graphite	*R*_int_ = 0.013
ω scans	θ_max_ = 28.3°, θ_min_ = 2.4°
Absorption correction: multi-scan (*SADABS*; Sheldrick, 1997)	*h* = −9→9
*T*_min_ = 0.101, *T*_max_ = 0.371	*k* = −13→13
4957 measured reflections	*l* = −14→15

### Refinement

**Table:**

Refinement on *F*^2^	Primary atom site location: structure-invariant direct methods
Least-squares matrix: full	Secondary atom site location: difference Fourier map
*R*[*F*^2^ > 2σ(*F*^2^)] = 0.027	Hydrogen site location: inferred from neighbouring sites
*wR*(*F*^2^) = 0.064	H atoms treated by a mixture of independent and constrained refinement
*S* = 1.05	*w* = 1/[σ^2^(*F*_o_^2^) + (0.0432*P*)^2^ + 0.5801*P*] where *P* = (*F*_o_^2^ + 2*F*_c_^2^)/3
3504 reflections	(Δ/σ)_max_ < 0.001
175 parameters	Δρ_max_ = 2.27 e Å^−^^3^
1 restraint	Δρ_min_ = −1.00 e Å^−^^3^

### Special details

**Table:**

Geometry. All e.s.d.'s (except the e.s.d. in the dihedral angle between two l.s. planes) are estimated using the full covariance matrix. The cell e.s.d.'s are taken into account individually in the estimation of e.s.d.'s in distances, angles and torsion angles; correlations between e.s.d.'s in cell parameters are only used when they are defined by crystal symmetry. An approximate (isotropic) treatment of cell e.s.d.'s is used for estimating e.s.d.'s involving l.s. planes.
Refinement. Refinement of *F*^2^ against ALL reflections. The weighted *R*-factor *wR* and goodness of fit *S* are based on *F*^2^, conventional *R*-factors *R* are based on *F*, with *F* set to zero for negative *F*^2^. The threshold expression of *F*^2^ > σ(*F*^2^) is used only for calculating *R*-factors(gt) *etc*. and is not relevant to the choice of reflections for refinement. *R*-factors based on *F*^2^ are statistically about twice as large as those based on *F*, and *R*- factors based on ALL data will be even larger.

### Fractional atomic coordinates and isotropic or equivalent isotropic displacement parameters (Å^2^)

**Table:**

	*x*	*y*	*z*	*U*_iso_*/*U*_eq_	
Au1	0.00874 (2)	0.215338 (16)	0.234396 (14)	0.01707 (7)	
Cl1	0.03040 (19)	−0.00191 (12)	0.22763 (11)	0.0318 (3)	
Cl2	−0.26390 (17)	0.12764 (11)	0.05577 (10)	0.0235 (2)	
Cl3	−0.00788 (18)	0.43396 (11)	0.24088 (10)	0.0249 (2)	
Cl4	0.27948 (17)	0.29983 (13)	0.41246 (10)	0.0287 (2)	
C5	0.6777 (7)	0.7714 (5)	0.9404 (4)	0.0215 (9)	
H5	0.6992	0.8678	0.9629	0.026*	
N6	0.5448 (6)	0.6896 (4)	0.8362 (3)	0.0207 (8)	
H6	0.477 (7)	0.718 (5)	0.778 (4)	0.025*	
C7	0.5351 (7)	0.5514 (4)	0.8204 (4)	0.0178 (8)	
C8	0.4115 (7)	0.4415 (5)	0.7204 (4)	0.0233 (9)	
H8	0.3166	0.4541	0.6517	0.028*	
C9	0.4313 (8)	0.3142 (5)	0.7244 (4)	0.0276 (10)	
H9	0.3487	0.2370	0.6571	0.033*	
C10	0.5711 (8)	0.2954 (5)	0.8257 (5)	0.0276 (10)	
H10	0.5811	0.2056	0.8254	0.033*	
C11	0.6940 (7)	0.4035 (5)	0.9253 (4)	0.0231 (9)	
H11	0.7888	0.3903	0.9936	0.028*	
C12	0.6738 (6)	0.5330 (4)	0.9220 (4)	0.0182 (8)	
S13	0.80728 (17)	0.68998 (12)	1.03100 (10)	0.0219 (2)	
O14	0.3180 (5)	0.7755 (3)	0.6536 (3)	0.0219 (7)	
C15	0.3808 (7)	0.9178 (5)	0.6497 (4)	0.0227 (9)	
H15B	0.5216	0.9468	0.6520	0.027*	
H15A	0.3715	0.9791	0.7195	0.027*	
C16	0.2358 (8)	0.9226 (5)	0.5300 (5)	0.0286 (10)	
H16B	0.2955	1.0014	0.5009	0.034*	
H16A	0.1068	0.9286	0.5372	0.034*	
C17	0.2060 (9)	0.7850 (5)	0.4458 (5)	0.0335 (12)	
H17A	0.0746	0.7534	0.3772	0.040*	
H17B	0.3159	0.7918	0.4127	0.040*	
C18	0.2116 (7)	0.6889 (5)	0.5298 (4)	0.0241 (9)	
H18B	0.0726	0.6352	0.5198	0.029*	
H18A	0.2830	0.6236	0.5108	0.029*	

### Atomic displacement parameters (Å^2^)

**Table:**

	*U*^11^	*U*^22^	*U*^33^	*U*^12^	*U*^13^	*U*^23^
Au1	0.01437 (9)	0.01940 (10)	0.01510 (9)	0.00527 (6)	0.00270 (6)	0.00332 (6)
Cl1	0.0340 (7)	0.0253 (6)	0.0289 (6)	0.0156 (5)	−0.0022 (5)	0.0042 (5)
Cl2	0.0220 (5)	0.0200 (5)	0.0205 (5)	0.0076 (4)	−0.0028 (4)	0.0022 (4)
Cl3	0.0284 (6)	0.0196 (5)	0.0221 (5)	0.0062 (4)	0.0045 (4)	0.0038 (4)
Cl4	0.0211 (5)	0.0370 (6)	0.0194 (5)	0.0097 (5)	−0.0013 (4)	0.0004 (5)
C5	0.025 (2)	0.019 (2)	0.023 (2)	0.0104 (18)	0.0086 (19)	0.0063 (18)
N6	0.0214 (19)	0.0210 (19)	0.0190 (18)	0.0094 (16)	0.0037 (15)	0.0059 (15)
C7	0.017 (2)	0.020 (2)	0.016 (2)	0.0064 (17)	0.0048 (17)	0.0062 (17)
C8	0.024 (2)	0.025 (2)	0.015 (2)	0.0063 (19)	0.0024 (18)	0.0030 (18)
C9	0.034 (3)	0.020 (2)	0.020 (2)	0.007 (2)	0.002 (2)	−0.0006 (18)
C10	0.034 (3)	0.021 (2)	0.026 (2)	0.013 (2)	0.005 (2)	0.0068 (19)
C11	0.025 (2)	0.021 (2)	0.022 (2)	0.0102 (19)	0.0023 (18)	0.0085 (18)
C12	0.014 (2)	0.020 (2)	0.017 (2)	0.0045 (17)	0.0016 (16)	0.0039 (17)
S13	0.0206 (5)	0.0221 (5)	0.0177 (5)	0.0071 (4)	0.0009 (4)	0.0025 (4)
O14	0.0250 (17)	0.0173 (15)	0.0198 (16)	0.0060 (13)	0.0031 (13)	0.0058 (12)
C15	0.025 (2)	0.020 (2)	0.024 (2)	0.0075 (19)	0.0089 (19)	0.0080 (18)
C16	0.035 (3)	0.025 (2)	0.028 (3)	0.013 (2)	0.009 (2)	0.010 (2)
C17	0.041 (3)	0.027 (3)	0.025 (3)	0.007 (2)	0.005 (2)	0.007 (2)
C18	0.023 (2)	0.025 (2)	0.020 (2)	0.0081 (19)	0.0012 (18)	0.0032 (18)

### Geometric parameters (Å, °)

**Table:**

Au1—Cl4	2.2733 (11)	C11—C12	1.390 (6)
Au1—Cl1	2.2835 (12)	C11—H11	0.9500
Au1—Cl2	2.2850 (11)	C12—S13	1.741 (4)
Au1—Cl3	2.2864 (11)	O14—C15	1.443 (5)
C5—N6	1.310 (6)	O14—C18	1.450 (5)
C5—S13	1.686 (4)	C15—C16	1.513 (6)
C5—H5	0.9500	C15—H15B	0.9900
N6—C7	1.392 (6)	C15—H15A	0.9900
N6—H6	0.86 (5)	C16—C17	1.525 (7)
C7—C8	1.387 (6)	C16—H16B	0.9900
C7—C12	1.398 (6)	C16—H16A	0.9900
C8—C9	1.368 (7)	C17—C18	1.518 (7)
C8—H8	0.9500	C17—H17A	0.9900
C9—C10	1.402 (7)	C17—H17B	0.9900
C9—H9	0.9500	C18—H18B	0.9900
C10—C11	1.373 (7)	C18—H18A	0.9900
C10—H10	0.9500		
			
Cl4—Au1—Cl1	90.14 (4)	C11—C12—S13	128.7 (3)
Cl4—Au1—Cl2	179.27 (4)	C7—C12—S13	110.4 (3)
Cl1—Au1—Cl2	89.16 (4)	C5—S13—C12	90.5 (2)
Cl4—Au1—Cl3	89.45 (4)	C15—O14—C18	109.0 (3)
Cl1—Au1—Cl3	179.07 (4)	O14—C15—C16	104.8 (4)
Cl2—Au1—Cl3	91.25 (4)	O14—C15—H15B	110.8
N6—C5—S13	114.0 (3)	C16—C15—H15B	110.8
N6—C5—H5	123.0	O14—C15—H15A	110.8
S13—C5—H5	123.0	C16—C15—H15A	110.8
C5—N6—C7	114.4 (4)	H15B—C15—H15A	108.9
C5—N6—H6	124 (4)	C15—C16—C17	101.8 (4)
C7—N6—H6	121 (4)	C15—C16—H16B	111.4
C8—C7—N6	127.8 (4)	C17—C16—H16B	111.4
C8—C7—C12	121.4 (4)	C15—C16—H16A	111.4
N6—C7—C12	110.8 (4)	C17—C16—H16A	111.4
C9—C8—C7	117.5 (4)	H16B—C16—H16A	109.3
C9—C8—H8	121.3	C18—C17—C16	102.9 (4)
C7—C8—H8	121.3	C18—C17—H17A	111.2
C8—C9—C10	121.4 (5)	C16—C17—H17A	111.2
C8—C9—H9	119.3	C18—C17—H17B	111.2
C10—C9—H9	119.3	C16—C17—H17B	111.2
C11—C10—C9	121.7 (4)	H17A—C17—H17B	109.1
C11—C10—H10	119.2	O14—C18—C17	106.7 (4)
C9—C10—H10	119.2	O14—C18—H18B	110.4
C10—C11—C12	117.2 (4)	C17—C18—H18B	110.4
C10—C11—H11	121.4	O14—C18—H18A	110.4
C12—C11—H11	121.4	C17—C18—H18A	110.4
C11—C12—C7	120.9 (4)	H18B—C18—H18A	108.6
			
S13—C5—N6—C7	0.3 (5)	N6—C7—C12—C11	−178.5 (4)
C5—N6—C7—C8	−179.6 (5)	C8—C7—C12—S13	179.4 (4)
C5—N6—C7—C12	−0.3 (5)	N6—C7—C12—S13	0.1 (5)
N6—C7—C8—C9	178.7 (5)	N6—C5—S13—C12	−0.2 (4)
C12—C7—C8—C9	−0.5 (7)	C11—C12—S13—C5	178.6 (4)
C7—C8—C9—C10	0.0 (8)	C7—C12—S13—C5	0.0 (4)
C8—C9—C10—C11	0.1 (9)	C18—O14—C15—C16	24.2 (5)
C9—C10—C11—C12	0.2 (8)	O14—C15—C16—C17	−37.1 (5)
C10—C11—C12—C7	−0.6 (7)	C15—C16—C17—C18	35.6 (5)
C10—C11—C12—S13	−179.0 (4)	C15—O14—C18—C17	−1.1 (5)
C8—C7—C12—C11	0.8 (7)	C16—C17—C18—O14	−22.0 (5)

### Hydrogen-bond geometry (Å, °)

**Table:**

*D*—H···*A*	*D*—H	H···*A*	*D*···*A*	*D*—H···*A*
N6—H6···O14	0.86 (5)	1.87 (5)	2.728 (5)	177 (6)
C5—H5···Cl2^i^	0.95	2.65	3.588 (5)	170
C8—H8···Cl4	0.95	2.93	3.447 (5)	116
C9—H9···Cl4	0.95	3.00	3.498 (5)	114
C10—H10···Cl2^ii^	0.95	2.96	3.541 (6)	121
C11—H11···Cl2^ii^	0.95	2.90	3.498 (5)	122
C11—H11···Cl3^ii^	0.95	2.77	3.639 (5)	154
C15—H15B···Cl1^iii^	0.99	3.02	3.922 (6)	153
C18—H18A···Cl4^iii^	0.99	2.91	3.547 (6)	123

Symmetry codes:  (i) *x*+1, *y*+1, *z*+1; (ii) *x*+1, *y*, *z*+1; (iii) −*x*+1, −*y*+1, −*z*+1.
